# Patient-Related Factors Associated with Mechanical Failure After Hemilaminectomy with Posterolateral Fusion: An Exploratory Retrospective Cohort Study †

**DOI:** 10.3390/healthcare14091199

**Published:** 2026-04-29

**Authors:** Oğuzhan Çiçek, Burak Keklikçioğlu, Hakan Uslu, İsmail Akçay, Ziya Çay, Osman Çiloğlu, Fırat Seyfettinoğlu, Evren Karaali

**Affiliations:** Department of Orthopedics and Traumatology, Adana City Training and Research Hospital, University of Health Sciences, Adana 01230, Turkey; drburakkeklikcioglu@gmail.com (B.K.); hakanusl77@gmail.com (H.U.); ismailakcay010@gmail.com (İ.A.); ziya.cay@hotmail.com (Z.Ç.); osmanciloglu@gmail.com (O.Ç.); firatseyf@yahoo.com (F.S.); drevrenkaraali@gmail.com (E.K.)

**Keywords:** lumbar spinal stenosis, hemilaminectomy, posterolateral fusion, implant-related mechanical failure

## Abstract

**Background**: Implant-related mechanical failure remains a clinically relevant concern following posterior decompression and fusion in elderly patients with lumbar spinal stenosis (LSS). The relative contribution of host-related versus construct-related factors to failure risk requires further clarification. **Methods**: This retrospective single-center cohort study included 118 patients aged ≥65 years who underwent single-level hemilaminectomy with posterolateral fusion (PLF) for isolated L4–5 central LSS, with a minimum follow-up of 48 months (mean 51.0 ± 2.0 months). All procedures were performed using a standardized posterior technique with uniform 6.5-mm titanium rods and 6.5-mm pedicle screws. Mechanical failure was defined as revision surgery due to radiographically and clinically confirmed hardware-related complications in the absence of infection. Exploratory univariable analyses were conducted to evaluate associations between baseline variables and mechanical failure. Clinical outcomes were assessed using validated patient-reported outcome measures. The Oswestry Disability Index (ODI), Roland Morris Disability Questionnaire (RMDQ), and Visual Analog Scale (VAS) for pain were recorded. **Results**: Overall revision rate was 13.6% (16/118), including 14 cases (11.9%) of implant-related mechanical failure and 2 cases (1.7%) of infection-related revision. Higher age (*p* = 0.005), higher body mass index (BMI) (*p* = 0.005), lower bone mineral density (BMD) (*p* < 0.001), active smoking (*p* < 0.001), and diabetes mellitus (DM) (*p* = 0.023) were significantly associated with mechanical failure. Functional outcomes (ODI, RMDQ, VAS) improved significantly at final follow-up (all *p* < 0.001). **Conclusions**: Mechanical failure following hemilaminectomy with PLF appears to be predominantly influenced by host-related factors rather than construct characteristics when a standardized surgical technique is applied. Bone quality and modifiable systemic risk factors may play a critical role in long-term construct durability.

## 1. Introduction

Posterolateral fusion (PLF) following posterior decompression continues to be applied as a surgical strategy in the management of lumbar spinal stenosis (LSS), particularly in elderly patients [1,2]. In selected cases, hemilaminectomy combined with PLF allows adequate neural decompression while preserving contralateral posterior elements and limiting surgical invasiveness [3]. By maintaining posterior tension band integrity and avoiding extensive bilateral decompression, this approach aims to balance neural decompression with segmental stability. Nevertheless, despite generally satisfactory clinical outcomes, mechanical complications related to implant failure remain a clinically relevant cause of revision surgery following hemilaminectomy with PLF [4,5].

From a biomechanical standpoint, hemilaminectomy with PLF represents a posterior-only construct in which spinal stability relies predominantly on pedicle screw fixation and the integrity of the posterior elements [6]. In contrast to interbody fusion techniques, anterior column support is not restored, and axial and cantilever loads are primarily transferred through posterior instrumentation and the quality of the surrounding bone [7,8]. In elderly patients, age-related reductions in bone mineral density (BMD), altered load-sharing patterns after unilateral decompression, and increased mechanical demands on posterior implants may compromise fixation strength and predispose constructs to implant-related mechanical failure [9,10,11].

Previous studies evaluating complications after lumbar fusion have frequently focused on postoperative infection or have compared different surgical techniques across heterogeneous patient populations, often including bilateral decompression or interbody fusion constructs [12,13]. Such heterogeneity limits the ability to isolate patient-related contributors to mechanical failure specifically within a hemilaminectomy and PLF construct. Importantly, implant-related mechanical failure is typically multifactorial and may reflect the interaction between biological factors, such as bone quality, and mechanical loading conditions inherent to posterior-only stabilization, rather than technical deficiencies of the implant itself [14,15,16].

Given that both the extent of decompression (hemilaminectomy) and the implant configuration (PLF) are uniform in this surgical approach, variability in mechanical outcomes is more likely driven by patient-related factors rather than surgical technique [3]. Identifying patient characteristics associated with mechanical failure may therefore provide clinically relevant insight for preoperative risk assessment and surgical planning, particularly in elderly individuals with compromised bone quality undergoing limited posterior decompression.

The purpose of this study was to evaluate clinical outcomes, revision patterns, and patient-related factors associated with implant-related mechanical failure in a homogeneous cohort of patients undergoing hemilaminectomy combined with PLF for isolated L4–5 LSS. By focusing on a posterior-only fusion construct with limited unilateral decompression and performing exploratory univariable analyses, this study seeks to generate biomechanically grounded hypotheses that may inform future prospective investigations and optimization strategies.

## 2. Methods

### 2.1. Study Design and Patient Selection

This study was designed as a retrospective cohort study conducted at a single tertiary referral center. Institutional ethics committee approval was obtained prior to data collection. Elderly patients who underwent hemilaminectomy combined with PLF for LSS were retrospectively reviewed.

**Figure 1:**
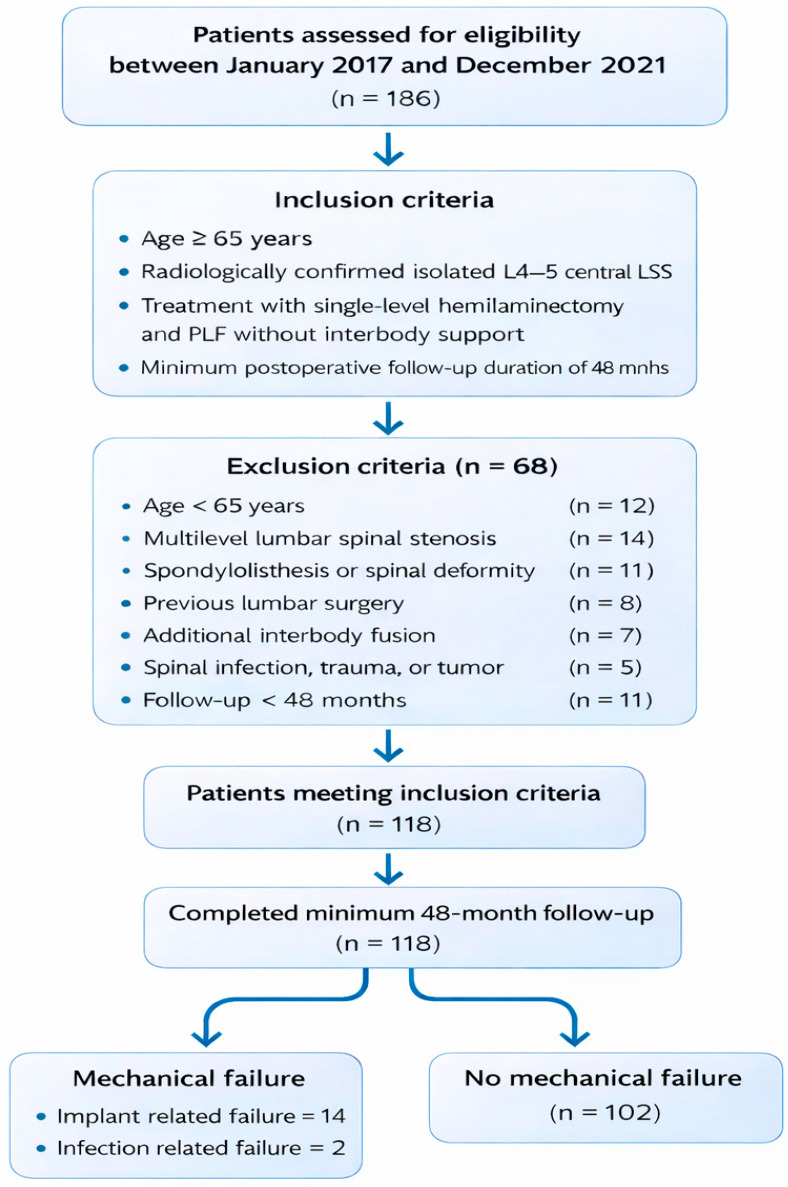
Flowchart of patient selection and study design.

Patients treated between January 2017 and December 2021 were screened. Inclusion criteria were age ≥ 65 years, radiologically confirmed isolated L4–5 central LSS, treatment with single-level hemilaminectomy and PLF without interbody support, and a minimum postoperative follow-up duration of 48 months. To ensure a homogeneous cohort and minimize biomechanical confounding, patients with spondylolisthesis, spinal deformity, multilevel stenosis, previous lumbar surgery, spinal infection, trauma, tumor, or additional interbody fusion were excluded. The patient inclusion and exclusion criteria and the overall study flow are presented in the flowchart in Figure 1.

### 2.2. Surgical Technique

All surgical procedures were performed by the same surgical team using a standardized posterior midline approach. Following decompression with hemilaminectomy, pedicle screw instrumentation was applied at the affected level, and PLF was performed using locally harvested autograft bone. Rods were contoured to restore segmental alignment and secured bilaterally. Titanium rods (6.5 mm) and pedicle screws (6.5 mm) were used uniformly across the cohort. No cement augmentation or expandable screw systems were used in this cohort.

Perioperative antibiotic prophylaxis consisted of intravenous cefazolin administered within 30 min prior to skin incision and continued for 24 h postoperatively. In patients with documented beta-lactam allergy, clindamycin was administered according to institutional protocol. A closed-suction drain was placed routinely and removed within 24–48 h unless clinical conditions required prolonged drainage.

Postoperatively, patients were mobilized on the first postoperative day under supervision. A standardized rehabilitation protocol emphasizing early ambulation and gradual return to daily activities was applied uniformly across the cohort. No routine external bracing was prescribed. Surgical and postoperative management strategies were consistent throughout the study period to minimize technique-related variability.

### 2.3. Clinical Outcome Measures

Clinical outcomes were assessed using validated patient-reported outcome measures. The Oswestry Disability Index (ODI), Roland Morris Disability Questionnaire (RMDQ), and Visual Analog Scale (VAS) for pain were recorded preoperatively and at the final follow-up visit.

ODI is a widely used instrument for evaluating functional disability related to low back pain. It consists of 10 sections, each scored from 0 to 5, with total scores expressed as percentages ranging from 0 to 100, where higher scores indicate greater disability.

RMDQ is a validated, self-administered questionnaire comprising 24 items that assess functional limitation associated with low back pain. Each affirmative response contributes one point, yielding a total score ranging from 0 to 24, with higher scores indicating greater disability.

VAS was used to quantify pain intensity on a continuous scale ranging from 0 (no pain) to 10 (worst imaginable pain).

### 2.4. Revision Surgery and Outcome Definitions

Revision surgery was defined as any unplanned reoperation performed at the index spinal level after the initial hemilaminectomy and PLF procedure. Revision surgeries were categorized according to etiology as implant-related mechanical failure or infection-related revision.

Implant-related mechanical failure included revision procedures performed for hardware-related complications such as screw loosening, rod breakage, or construct instability in the absence of clinical or laboratory evidence of infection. Infection-related revision was defined as reoperation required for deep postoperative infection based on clinical findings, laboratory parameters, imaging studies, and intraoperative assessment. Mechanical failure was defined based on both radiological and clinical criteria. Radiographic evidence included progressive radiolucent zones around pedicle screws, implant migration, rod fracture, loss of segmental alignment, or signs of construct instability observed on standing radiographs and confirmed by computed tomography when necessary. Pseudarthrosis was considered when there was absence of solid fusion accompanied by hardware loosening or persistent mechanical back pain requiring revision surgery. Only cases requiring surgical intervention were classified as implant-related mechanical failure events. Accordingly, asymptomatic or conservatively managed pseudarthrosis cases were not captured within the failure endpoint.

### 2.5. Baseline Variables and Risk Factors

Baseline demographic and clinical variables included age, sex, body mass index (BMI), smoking status, and presence of diabetes mellitus (DM). DM was recorded as a physician-diagnosed condition documented in institutional medical records at the time of surgery; no stratification based on glycemic control parameters was performed. Smoking status was defined as active tobacco use at the time of surgery, and former smokers who had discontinued smoking at least 12 months prior to surgery were not classified as active smokers. BMD was assessed using dual-energy X-ray absorptiometry (DEXA), and T-scores were recorded as continuous variables. Perioperative variables (surgical duration, estimated blood loss, and length of hospital stay) were included for descriptive purposes and exploratory comparisons but were not part of the primary analytical framework. Follow-up duration was calculated in months from the index surgery to the most recent clinical evaluation.

### 2.6. Statistical Analysis

Descriptive statistics were used to summarize baseline characteristics and clinical outcomes. Continuous variables are presented as mean ± standard deviation or median with interquartile range, as appropriate, while categorical variables are reported as number and percentage. Preoperative and final follow-up ODI, RMDQ, and VAS scores were compared using paired statistical testing.

Exploratory univariable analyses were performed to evaluate associations between patient-related factors and implant-related mechanical failure. Continuous variables were compared using non-parametric methods, and categorical variables were analyzed using exact tests. A total of 14 mechanical failure events were observed; with 6 candidate predictors, the events-per-variable ratio was approximately 2.3, which is below commonly recommended thresholds for reliable multivariable modeling. Accordingly, all analyses were interpreted as hypothesis-generating. Statistical significance was set at *p* < 0.05.

Detailed radiographic alignment parameters were not included, as the primary focus of the study was implant-related mechanical failure rather than sagittal balance correction.

## 3. Results

### 3.1. Patient Characteristics

A total of 118 patients who underwent hemilaminectomy combined with PLF for isolated L4–5 LSS were included in the final analysis. Baseline demographic characteristics, comorbidities, BMD values, and perioperative variables are summarized in Table 1. All patients met the minimum follow-up requirement of 48 months.

No intraoperative complications were recorded. Perioperative parameters, including surgical duration, estimated blood loss, length of hospitalization, and time to return to work, are presented descriptively in Table 1.

### 3.2. Clinical Outcomes

Significant improvements were observed in all patient-reported outcome measures at final follow-up compared with preoperative values. Changes in the ODI, RMDQ, and VAS scores are detailed in Table 2. Within-group comparisons demonstrated statistically significant postoperative improvement across all clinical outcome measures.

### 3.3. Revision Surgery Profile

Revision surgery was required in a subset of patients during the follow-up period. Implant-related mechanical failure represented the predominant indication for revision, whereas infection-related revision was observed less frequently. The distribution and etiology of revision surgeries are summarized in Table 2.

### 3.4. Exploratory Univariable Analyses for Implant-Related Mechanical Failure

Exploratory univariable analyses were performed to evaluate associations between patient-related factors and implant-related mechanical failure. Comparisons between patients with and without mechanical failure are presented in Table 3.

**Figure 2:**
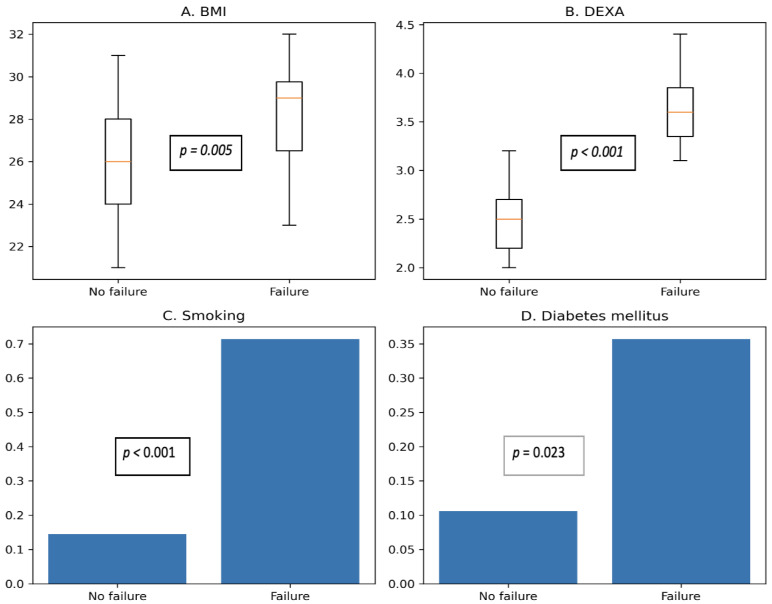
Distribution of key patient-related risk factors associated with implant-related mechanical failure.

Higher age, higher BMI, lower BMD, smoking, and the presence of DM were significantly associated with implant-related mechanical failure. Sex was not significantly associated with mechanical failure. Continuous and categorical variables were analyzed using appropriate non-parametric and exact statistical methods, as outlined in the Section 2. Detailed statistical results are provided in Table 3 and Figure 2.

## 4. Discussion

This study demonstrates that implant-related mechanical failure following hemilaminectomy combined with PLF is predominantly associated with patient-related biomechanical and biological factors rather than implant design or technical variability. Within a homogeneous cohort treated using a standardized posterior-only PLF construct, chronological age, reduced BMD, increased BMI, the presence of DM and smoking were associated with mechanical failure, whereas sex showed no significant association with mechanical failure. Although chronological age and DM demonstrated significant associations in univariable analysis, these findings should be interpreted in light of their close associations with bone quality and metabolic factors. Importantly, these factors should not be interpreted as isolated determinants; rather, they appear to represent an interrelated cluster of host-related biological and mechanical vulnerability. From a clinical perspective, these findings underscore the importance of preoperative patient optimization. Strategies such as systematic evaluation and treatment of low bone mineral density, smoking cessation, and optimization of glycemic control in patients with diabetes may improve construct stability and reduce the risk of mechanical failure in posterior-only fusion procedures.

All patients in the present cohort underwent the same surgical approach with identical implant constructs, thereby minimizing variability related to surgical technique or instrumentation. This construct uniformity strengthens the interpretation that mechanical failure in PLF reflects a biomechanical mismatch between patient characteristics and construct load-sharing capacity, rather than isolated implant-related deficiencies [3]. Accordingly, implant-related mechanical complications should be interpreted as the consequence of patient-specific biological vulnerability interacting with the mechanical demands imposed by posterior-only stabilization.

From a biomechanical perspective, these findings are consistent with the fundamental load-sharing characteristics of PLF constructs [6,7]. In contrast to interbody fusion techniques, PLF provides limited anterior column support and relies predominantly on posterior instrumentation and the integrity of the posterior elements to maintain segmental stability [8]. As a result, axial loads and bending moments are preferentially transmitted through the pedicle screw–rod construct and the adjacent bone–implant interface, thereby increasing mechanical demands on posterior fixation [9,10]. In the presence of compromised bone quality, this loading pattern may exceed local fixation capacity and predispose constructs to screw loosening, rod deformation, or global mechanical failure [11,17]. In addition, sagittal alignment parameters, including pelvic incidence–lumbar lordosis mismatch, are known to significantly influence load distribution across posterior constructs, and their contribution to mechanical complications has been emphasized in recent studies on spinal deformity and junctional failure [18].

The observed association between lower DEXA T-scores and implant-related mechanical failure underscores the central importance of bone quality in posterior-only fusion constructs. Reduced trabecular density weakens pedicle screw purchase and diminishes resistance to repetitive cyclic loading, a vulnerability that is accentuated in the absence of anterior column load sharing [17,19]. Notably, this relationship persisted independent of chronological age, supporting the concept that intrinsic bone quality rather than age alone is a dominant determinant of mechanical stability in PLF constructs.

Higher BMI was also associated with mechanical failure, likely reflecting increased axial loading and cantilever forces acting across the instrumented segment [20]. In posterior-only constructs such as PLF, elevated BMI amplifies repetitive mechanical stresses at the bone–implant interface, promoting micromotion and accelerating fatigue-related failure of posterior instrumentation. The concurrent association between smoking and mechanical failure further emphasizes the contribution of biological factors, as smoking adversely affects bone metabolism, vascular supply, and fusion biology, thereby reducing the capacity of the bone–implant interface to tolerate sustained mechanical loading [21,22]. Although a 12-month cessation threshold was applied, the cumulative effects of tobacco use may not be fully reversible, and residual biological impact may persist. Therefore, the influence of smoking on mechanical failure may still be underestimated.

DM emerged as an additional patient-related factor associated with implant-related mechanical failure. This association is biologically plausible and supported by established evidence linking diabetes to impaired bone metabolism, microvascular dysfunction, and delayed bone remodeling [23,24]. In posterior-only constructs such as PLF—where stability depends largely on pedicle screw fixation and surrounding bone integrity—diabetes-related alterations may further reduce mechanical tolerance and increase susceptibility to construct failure under repetitive loading conditions. However, diabetes was recorded as a physician-diagnosed condition without stratification by glycemic control (e.g., HbA1c levels); therefore, the observed association should be interpreted with caution.

These findings have direct implications for surgical decision-making. In patients with markedly reduced BMD or increased BMI, the posterior-only load-sharing characteristics of PLF may provide insufficient mechanical reserve, making strategies that enhance fixation strength or augment anterior column support reasonable considerations [25]. Conversely, when bone quality and biomechanical demands are favorable, hemilaminectomy combined with PLF remains an effective and balanced option, offering adequate stability while limiting surgical invasiveness.

Several limitations warrant consideration. The retrospective design and single-center setting may limit generalizability. Although follow-up duration was sufficient to detect clinically relevant mechanical failure events, only 14 revision cases were observed. With this event rate, multivariable regression modeling was considered statistically unstable due to an insufficient events-per-variable ratio and a high risk of overfitting. As a result, potential confounding among interrelated variables such as age, BMI, diabetes mellitus, smoking, and bone mineral density (BMD) cannot be ruled out, and the findings should be interpreted as exploratory and hypothesis-generating rather than causal. In addition, certain perioperative and biomechanical factors that may influence construct longevity were not available for analysis. The absence of a standardized, validated fusion grading system (e.g., Bridwell classification or CT-based criteria) represents a limitation of this study; however, fusion status was assessed based on consistent clinical and radiographic findings, including evaluation of trabecular continuity and hardware integrity, with CT imaging used in cases of diagnostic uncertainty. Preoperative sagittal alignment parameters were not systematically assessed; although patients with overt deformity and spondylolisthesis were excluded to reduce major biomechanical confounding, the absence of detailed spinopelvic evaluation may represent a residual mechanical blind spot influencing construct loading. Despite these limitations, the homogeneous cohort, standardized surgical technique with uniform implant characteristics, and consistent follow-up protocol strengthen the internal validity of the findings.

## 5. Conclusions

In conclusion, implant-related mechanical failure following hemilaminectomy with PLF for single-level L4–5 LSS appears to be predominantly influenced by patient-specific factors rather than construct-related variables when a standardized surgical technique and uniform implant system are employed. Lower BMD, active smoking, and metabolic comorbidities were associated with mechanical failure in exploratory analyses. These findings suggest that mechanical durability in elderly spine patients is closely linked to underlying bone quality and systemic health rather than solely to surgical execution. Accordingly, preoperative risk stratification and optimization—including evaluation of BMD and modification of modifiable risk factors—may represent critical steps in reducing construct-related complications. Given the limited number of failure events and the exploratory nature of the statistical modeling, prospective, adequately powered multicenter studies are warranted to validate these associations and to refine patient-specific surgical planning strategies in this population.

## Figures and Tables

**Table 1:** Baseline Demographic Characteristics of the Hemilaminectomy + Posterolateral Fusion Cohort (n = 118).

Variable	Hemi + PLF Cohort
Age, years (mean ± SD)	68.1 ± 3.2
Female sex, n (%)	76 (64.4)
Male sex, n (%)	42 (35.6)
Body mass index, kg/m^2^ (mean ± SD)	26.2 ± 2.7
Diabetes mellitus, n (%)	16 (13.6)
Smoking, n (%)	26 (22.0)
DEXA T-score (mean ± SD)	−2.59 ± 0.49
Follow-up duration, months (mean ± SD)	51.0 ± 2.0
Surgical duration (min)	108 ± 28.1
Blood loss (cc)	252.2 ± 118.0
Hospitalization (days)	3.18 ± 1.1
Return to work (days)	103.1 ± 10.3

Values are presented as mean ± standard deviation or number (percentage), as appropriate.

**Table 2:** Clinical Outcomes and Revision Profile of the Hemilaminectomy + Posterolateral Fusion Cohort.

Outcome Measure	Preoperative	Final Follow-Up	*p*-Value
ODI	32.22 ± 2.0	16.84 ± 1.4	<0.001
RMDQ	18.3 ± 3.1	6.9 ± 2.7	<0.001
VAS	7.40 ± 1.8	2.85 ± 1.2	<0.001
Any revision, n (%)	–	16 (13.6)	–
Infection-related revision, n (%)	–	2 (1.7)	–
Implant-related mechanical failure, n (%)	–	14 (11.9)	–

ODI: Oswestry Disability Index; VAS: Visual Analogue Scale; RMDQ: Roland Morris Disability Questionnaire. Continuous outcomes were compared using paired statistical testing. Revision outcomes are reported descriptively as number (percentage).

**Table 3:** Exploratory Univariable Factors Associated with Implant-Related Mechanical Failure After Hemilaminectomy + Posterolateral Fusion.

Variable	No Mechanical Failure	Mechanical Failure	*p*-Value
Age, years	67.0 (IQR 66.0–69.0)	71.0 (IQR 68.0–72.8)	0.005
BMI, kg/m^2^	26.0 (IQR 24.0–28.0)	29.0 (IQR 26.5–29.8)	0.005
DEXA T-score	−2.5 (IQR −2.7 to −2.2)	−3.6 (IQR −3.9 to −3.3)	<0.001
BMD category			0.015
Normal	6 (5.9)	0 (0)	
Osteopenia (−1.0 to −2.5)	38 (37.3)	1 (7.1)	
Osteoporosis (≤−2.5)	58 (56.9)	13 (92.9)	
Diabetes mellitus, n (%)	11 (10.6)	5 (35.7)	0.023
Smoking, n (%)			<0.001
Yes	15 (14.7)	11 (78.6)	
No	87 (85.3)	3 (21.4)	
Sex, n (%)			0.580
Male	36 (34.8)	6 (42.9)	
Female	66 (64.2)	8 (57.1)	
Surgical duration (min)	105 (90–120)	110 (95–130)	0.396
Blood loss (mL)	240 (180–300)	260 (200–320)	0.471
Length of hospital stay (days)	3 (2–4)	3 (3–4)	0.334

Footnote: Continuous variables are presented as median (interquartile range), and categorical variables as number (percentage). Percentages are presented as column-wise proportions within each comparison group. Exploratory univariable analyses were performed using non-parametric methods for continuous variables and exact tests for categorical variables. All findings are hypothesis-generating. BMD = Bone Mineral Density.

## Data Availability

The datasets generated and/or analyzed during the current study will be made available by the corresponding author upon reasonable request. This approach has been adopted to ensure compliance with patient confidentiality and institutional ethical regulations.
